# Development and validation of the IneRISE-Iman model: a spiritual care learning framework for undergraduate nursing students

**DOI:** 10.1186/s12909-026-10079-z

**Published:** 2026-08-05

**Authors:** Inggriane Puspita Dewi, Hartiah Haroen, Hana Rizmadewi Agustina, Tuti Pahria, Nita Arisanti, Sharifah Shafinaz Sh Abdullah

**Affiliations:** 1 Doctoral Study Program in Medical Science, Faculty of Medicine, Padjadjaran University, Bandung, West Java Indonesia; 2 Department of Nursing, Faculty of Health Sciences, Universitas’ Aisyiyah, Bandung, West Java Indonesia; 3 Department of Community Nursing, Faculty of Nursing, Padjadjaran University, Bandung, West Java Indonesia; 4 Department of Basic Nursing, Faculty of Nursing, Padjadjaran University, Bandung, West Java Indonesia; 5 Department of Medical-Surgical Nursing, Faculty of Nursing, Padjadjaran University, Bandung, West Java Indonesia; 6 Department of Public Health, Padjadjaran University, Bandung, West Java Indonesia; 7 Centre for Nursing Studies, Universiti Teknologi Mara, Puncak Alam, Selangor Malaysia

**Keywords:** Spirituality, Delphi technique, Patient-centered care, Islam, Muslims, Education, Nursing, Curriculum

## Abstract

**Background:**

Integrating spiritual care into undergraduate nursing curricula remains inconsistent, particularly in Muslim-majority contexts. Existing models are predominantly grounded in Western or secular frameworks and fail to adequately address the theological and cultural dimensions of Islamic spiritual care. A validated, culturally responsive pedagogical framework is therefore needed.

**Aim:**

To develop and establish the content validity of the IneRISE-IMAN (*Islamic Nurturing Experience through Reflective-Islamic values embedded*,* Simulated and Engaged Learning for Internalization*,* Meaningful*,* Active Communication*,* and Nurturing*) model for undergraduate nursing education.

**Methods:**

A two-round electronic Delphi (e-Delphi) study was conducted with 22 purposively selected experts representing nursing education, clinical practice, Islamic chaplaincy, and nursing students. Content validity was assessed using I-CVI, S-CVI/Ave, and S-CVI/UA. Inter-rater agreement was examined using Fleiss’ Kappa.

**Results:**

Round 1 demonstrated excellent content validity (S-CVI/Ave = 0.94; S-CVI/UA = 79%). Following targeted revision of six sub-elements, full universal agreement was achieved in Round 2 (S-CVI/UA = 1.00), with 100% panel retention across both rounds. Fleiss’ Kappa indicated moderate inter-rater agreement (κ = 0.44), acceptable for an interdisciplinary panel.

**Conclusion:**

The IneRISE-IMAN model shows strong content validity as a theoretically grounded framework that integrates Islamic philosophical anthropology, constructivist learning theory, and person-centered nursing for Islamic spiritual care education in undergraduate nursing. Future research should explore construct validity, implementation feasibility, and the impact on students’ clinical skills.

**Supplementary Information:**

The online version contains supplementary material available at https://doi.org/10.1186/s12909-026-10079-z.

## Background

Across the contemporary nursing literature, spiritual care is widely framed as a vital element of holistic, person-centered healthcare [1–3]. Drawing on widely cited conceptualizations by Vincensi [1] and Puchalski [4], spirituality is understood in this literature as a dynamic, multidimensional dimension of human experience involving the search for meaning, purpose, connection, and transcendence, and spiritual care as the intentional recognition of, and response to, patients’ spiritual needs in order to promote their overall well-being [1, 5].

Spiritual care encompasses several aspects, including managing physical symptoms to support patients’ search for meaning, purpose, hope, and connection during illness, suffering, and vulnerability [1, 2]. In nursing practice, spirituality-oriented care has been associated with improved coping, emotional well-being, ethical sensitivity, clinical decision-making, and patient Satisfaction, especially in chronic illness, rehabilitation, and palliative care settings [4, 5]. Consequently, the International Nursing Board increasingly emphasizes integrating spiritual care competencies into undergraduate nursing curricula to prepare future nurses for compassionate and comprehensive practice [6–8].

Although spirituality is often described as a universal human dimension that transcends formal religious affiliation, empirical evidence consistently demonstrates that spiritual needs are frequently expressed through religious beliefs, rituals, moral values, and faith-informed interpretations of illness and healing [9, 10]. In Muslim-majority settings, such as Indonesia, spirituality is deeply connected with Islamic teachings, which include beliefs about divine decree (Qadar), trust in God (Tawakkul), patience during suffering (Sabr), hope (Raja’), moral duty, and community bonds [11, 12]. These Islamic teachings significantly influence how Muslim patients perceive illness, experience suffering, care for themselves, and interact with healthcare services.

It is therefore important to distinguish between generic spiritual care and Islamic spiritual care: while both share common humanistic values, the latter is grounded in Islamic theology, ethics, and specific religious practices, requiring culturally and religiously informed competencies that go beyond general spiritual care frameworks.

Despite its acknowledged importance, the integration of spiritual care into undergraduate nursing curricula remains inconsistent and underdeveloped, particularly in non-Western settings [9, 13]. This inconsistency represents a critical problem: without a structured and culturally grounded educational framework, nursing graduates in Muslim-majority countries may lack the competencies needed to deliver spiritually congruent care to their patients.

Existing curriculum initiatives related to spiritual care are often fragmented, variably implemented, and weakly grounded in explicit cultural, religious, or pedagogical frameworks. Furthermore, although numerous spiritual care education models exist internationally, most have been developed within Western or secular educational traditions and do not adequately account for the epistemological, theological, and cultural dimensions of Islamic spiritual care [14, 15].

Evidence from a recent systematic review focusing on undergraduate nursing curricula in the Middle East and Asia indicates that while structured learning content can enhance students’ spiritual awareness and competence, substantial gaps persist in curricular coherence and cultural contextualization. Notably, many of the reviewed studies originated from Muslim-majority countries; however, few explicitly integrated culturally sensitive approaches grounded in Islamic spiritual perspectives [16]. These gaps contribute to graduates’ uncertainty and limited confidence in delivering spiritually congruent care in clinical practice.

While generic, theoretically informed spiritual care competency frameworks have been developed and validated for pre-registration nursing, most notably the EPICC consensus standard and competency framework in Europe [8, 17, 18], and a growing body of scholarship has articulated the theological foundations of Islamic spiritual care [11, 12], these two streams of work have rarely been integrated. To date, there is no validated pedagogical model that specifically implements Islamic spiritual care within undergraduate nursing education. This model would need to integrate Islamic moral philosophy, culturally relevant spiritual concepts, and contextually appropriate teaching strategies into a single, content-validated educational framework. This gap is particularly significant in Indonesia, the largest Muslim-majority country in the world, where the practice of spiritual care is deeply embedded in Islamic beliefs. Yet there is currently no standardized educational framework to guide undergraduate nursing training in this important area.

## Theoretical framework of the IneRISE-IMAN model

The IneRISE-IMAN (Islamic Nurturing Experience through Reflective-Islamic Values Embedded, Simulated, and Engaged Learning for Internalization, Meaningful, Active Communication, and Nurturing) model is grounded in three complementary theoretical traditions that provide its ontological, epistemological, and praxis foundations. For this model, spirituality is defined as a dynamic, multidimensional human experience involving the search for meaning, purpose, transcendence, and connection with the divine [1, 4]. Spiritual care refers to the intentional, competency-based nursing practice of recognizing and responding to patients’ spiritual needs to support their well-being and dignity during illness and suffering [19]. Importantly, the model distinguishes Islamic spiritual care from secular and generic religious frameworks by incorporating universal nursing values, empathy, therapeutic presence, and person-centered practice with Islamic moral philosophy and faith-specific practices relevant to Muslim patients, such as trust in God (tawakkul), patience (sabr), hope (raja’), and remembrance of God (dhikr) [11, 12, 20]. This conceptual positioning ensures theoretical consistency and international readability across diverse nursing and Islamic healthcare contexts.

At the ontological level, the framework draws on Imam al-Ghazali’s theory of human nature, which views the human being as consisting of (body) jism, ego (nafs), heart (qalb), intellect (aql), and spirit (ruh), with true well-being (sa’ādah) achieved through purification of the heart (tazkiyatun nafs) [21]. This directly supports the I (Islamic values embedded) dimension of RISE and each IMAN outcome: Internalization reflects the integration of (knowledge) ‘ilm and (moral) akhlaq. Meaningful care aligns with the higher objectives of Islamic law (maqashid al-sharia). Active communication embodies Islamic ethics of sincerity (sidq) and gentleness (rifq), and nurturing interventions operationalize worship to God (ibadah) and service to humanity (Khidmah) [22, 23].

At the epistemological level, constructivist learning theory, synthesizing Piaget, Vygotsky, and Dewey, structures the RISE learning process [24]. Piaget’s schema adaptation informs the S (Simulated) dimension; Vygotsky’s Zone of Proximal Development supports the E (Engaged Learning) dimension through scaffolded peer collaboration and lecturer mentoring; Dewey’s reflective pragmatism guides the R (Reflective) dimension through structured reflection enriched by Islamic reflection (tafakkur and muhasabah) [25–27]; and Mezirow’s transformative learning anchors the I (Internalization) outcome of IMAN [28].

At the praxis level, McCormack and McCance’s Person-Centered Nursing Framework operationalizes spiritual understanding into clinical outcomes [29, 30]. Prerequisites map onto the E dimension through Uswah Hasanah (exemplary lecturer role modeling); the care environment corresponds to the S dimension; person-centered processes are expressed through A (Active Communication) and M (Meaningful) outcomes; and the PCN outcome domain is realized through N (Nurturing) interventions [1, 31].

Overall, al-Ghazali presents a teleological view, constructivism offers the learning framework, and person-centered nursing applies this in clinical practice, creating an educational model that is simultaneously Islamic in orientation, constructivist in pedagogy, and person-centered in practice.

## Methods

### Study design

This study employed a two-round electronic Delphi (e-Delphi) methodology to develop and establish the content validity of a spiritual care learning framework for undergraduate nursing. The Delphi method is widely used to achieve structured expert consensus in fields where empirical evidence is limited or theoretical constructs are complex, particularly in the development of educational models and competency frameworks [32]. The electronic format was selected to enable geographically distributed expert participation, preserve respondent anonymity, and minimize the influence of dominant opinions on group consensus [33].

The decision to conduct two rounds was based on three considerations. First, content validity studies using the Delphi method do not require consensus to be saturated in the same way as forecasting or policy Delphi designs; two rounds are generally sufficient when high item-level agreement is reached in the first round, and the remaining items reach consensus after targeted revision [34, 35]. Second, the structured quantitative criteria used in this study (I-CVI ≥ 0.78 for acceptance) provided an objective decision rule for resolving items, reducing the need for additional rounds due to ambiguity. Third, maintaining panelist engagement is clearly higher in shorter Delphi processes, supporting the validity of the second-round data [33].

These considerations collectively justify the two-round design as both methodologically appropriate and practically sound for a content validation study of this nature.

### Conceptual framework development

The development of the IneRISE-IMAN model was part of a larger, three-phase research program. In Phase I, a needs assessment was conducted through a systematic review and a qualitative descriptive exploratory study to identify gaps, expectations, and contextual requirements that informed model development [16]. Phase II: The focus of this study was to develop the initial conceptual framework and establish its content validity through two rounds of the e-Delphi method. Phase III will assess the model’s feasibility and initial impact via a feasibility study and a limited quasi-experimental design. The initial conceptual framework, based on Phase I findings and relevant theoretical literature, was organized into three preliminary domains: (1) learning inputs (2), learning processes, and (3) expected competency outcomes, presented in Table 1, which guided the structure of the Delphi questionnaire.

**Table 1:** Key elements and sub-elements learning model in Islamic spiritual care^a^

Sources	Elements input	Elements process	Elements outcomes
Theoretical framework	Person Centre Care and Human Nature-Soul 1. Therapeutic relationships grounded in patients’ values and meanings 2. Active involvement of patients and families in nursing care 3. A supportive nursing practice environment 4. Nursing care that addresses patients’ spiritual needs 5. *Tazkiyatun nafs* (self-purification)	Constructive Learning and Human Nature-Soul 1. Active, evidence-based learning 2. Collaborative learning 3. Reflective learning	*Human Nature-Soul* 1. *Qalbun Salīm* (sound heart) 2. Integration of knowledge, worship, and Islamic ethics and moral conduct (*akhlaq*)
Systematic review	1. Nursing students third to fourth years 2. Senior lecturer 3. Lesson plan (4 to 18 weeks program), 50–120 min for Classroom-based session 4. Material/module for theories and practice (spirituality and spiritual care, nursing process in spiritual care, moral ethics in spiritual care) 5. Video, e-book, movies, e-learning platform 6. Methods: students focus on learning (case study, role play, problem solving, simulated learning), classroom-based, and practicing in laboratory or clinical settings	1. Reflective learning (Gibbs) 2. Experiential learning (Kolb)	1. Spiritual care competencies 2. Self-efficacy in providing spiritual care 3. Caring behavior 4. Spiritual well-being among nursing students
Qualitative findings	1. Nursing students a. Characteristics b. Self-efficacy c. Learning style d. Academic resilience 2. Patients and family members a. Spiritual problem b. Moral-ethical problem c. Spiritual needs d. Social Needs 3. Curriculum a. Lesson plan b. Material (spirituality-spiritual care in Islamic context, nursing process in Islamic spiritual care, Islamic moral-ethics) c. Learning methods (Hybrid online-offline class, student-centered learning, evidence-based practice, case study based on students’ experience) d. Learning evaluation 4. Learning environment a. Peer group support and active discussion b. Islamic nuances 5. Lecturer a. Academic qualification b. Pedagogical competencies c. Role model d. Experiences 6. Facilities a. Classroom and laboratory b. Module c. Technological innovation	1. Reflective process a. Description (trigger and observation) b. Feeling (trigger) c. Evaluation d. Analysis e. Conclusion (synthesis) f. Action plan (transformation dan renew) 2. Writing/reflective journaling	Spiritual care competencies a. Internalization (Knowledge) b. Meaning (Knowledge and attitude) c. Active Communication (Psychomotor) d. Nurturing (Psychomotor)

^a^before the decision in the committee steering

### Development of the Delphi questionnaire

A structured questionnaire was developed based on the refined conceptual framework, comprising 71 items covering the model’s elements and sub-elements. Each item was rated on a 9-point Likert scale, with scores of 1–3 indicating low relevance, 4–6 indicating moderate relevance or uncertainty, and 7–9 indicating high relevance and importance. A 9-point scale was selected over a 5-point scale because it improves response discrimination and reduces central-tendency bias in content validity studies [35]. An open-ended comment section followed each item, enabling panelists to suggest revisions, provide contextual clarifications, and offer additional insights. This qualitative feedback contextualized the quantitative ratings and guided model refinement across rounds.

### Expert panel selection

#### Eligibility criteria

Inclusion criteria for panel membership were: (1) a minimum of five years of academic or professional experience in at least one of the following domains nursing education, clinical nursing practice, spiritual care, Islamic health sciences, or curriculum development; (2) demonstrated scholarly or professional contribution to the relevant field, evidenced by publications, teaching responsibilities, or clinical specialisation; and (3) willingness and availability to complete both rounds of the e-Delphi process within the designated timeframes. Chaplains with formal qualifications in Islamic spiritual care and final-year professional nursing students were additionally included to ensure the panel captured both expert theological perspectives and the learner’s viewpoint, a deliberate design decision to enhance the ecological validity of the validated framework.

#### Recruitment strategy

Consistent with established Delphi methodology, panelists were not sampled randomly but purposively selected for their substantive expertise, since the validity of a Delphi study depends on the relevance and quality of the participating experts rather than on statistical representativeness [32, 36, 37]. This expert-identification approach, supplemented by snowball referral, parallels the recruitment logic adopted in comparable spiritual care Delphi studies, including the competency framework of Attard et al. [17] and the consensus-based education standard of van Leeuwen et al. [6, 8]. Initial candidates were identified through three channels: (1) review of authorship in relevant peer-reviewed publications on spiritual care education in Muslim-majority contexts; (2) consultation with senior academics at Padjadjaran University and partner institutions; and (3) recommendations from key informants identified during Phase I of the broader research program. Identified candidates were contacted individually by e-mail and through established institutional and professional networks. Each prospective panelist received a formal written invitation together with a participant information sheet detailing the study’s purpose, procedures, estimated time commitment, the voluntary nature of participation, and ethical assurances of anonymity and confidentiality. Written informed consent was obtained electronically prior to Round 1, and ethical principles, namely voluntary participation, the right to withdraw without penalty, and protection of anonymity throughout the iterative rounds, were upheld in accordance with the institutional ethical approval granted for the study (No. 7/UN6.KEP/EC/2025).

#### Panel composition and size

A total of 25 experts were invited to participate in the Delphi study. Invitations were disseminated individually rather than through an open call: each candidate received a personalized e-mail containing the participant information sheet, the consent form, and a secure link to the online survey platform. Of these, 22 experts provided consent, completed Round 1, and were enrolled in the panel, resulting in an 88.0% response rate. Responses were automatically collected and stored by the survey platform under anonymized participant codes. This response rate exceeds the minimum level generally considered acceptable for Delphi studies and supports the representativeness and credibility of the expert panel. The participants represented diverse professional roles: nursing lecturers (including professors), clinical nurses, hospital chaplains, and professional nursing students. Participants were drawn from multiple universities and healthcare institutions across Indonesia, with one international representative from Malaysia, to enhance the contextual and institutional diversity of expert perspectives. Panel size was determined in accordance with recommendations that Delphi panels should be sufficiently large to represent the domain’s breadth while remaining manageable for iterative engagement; samples of 10–30 participants are considered appropriate for specialized content validation studies [36, 37].

#### Panel continuity

To maintain methodological rigor across rounds, all 22 panelists who completed Round 1 were invited to participate in Round 2. Panel continuity was monitored by the research team, and individualized follow-up reminders were sent to non-respondents within the first 48 h of the Round 2 deadline. Full panel retention was achieved across both rounds (response rate: 100%), ensuring that Round 2 consensus ratings reflected the same expert group that had provided the initial ratings and qualitative feedback.

### Delphi procedures

The e-Delphi process was administered via a secure online survey platform. Round 1 was open for two weeks, during which panelists independently rated each item on the 9-point scale and provided open-ended qualitative feedback. Anonymity was preserved throughout; panelists were not informed of each other’s identities or individual ratings. Following data collection, quantitative responses were analyzed descriptively, and item-level content validity indices (I-CVI) were calculated for each item. Items with an I-CVI of ≥ 0.78 were accepted without modification; items with an I-CVI between 0.70 and 0.77 were revised based on qualitative feedback; and items with an I-CVI below 0.70 were substantially restructured or removed [36]. All qualitative comments were compiled, thematically summarized by a trained research assistant, and reviewed by the research team to guide targeted revisions.

**Fig. 1:**
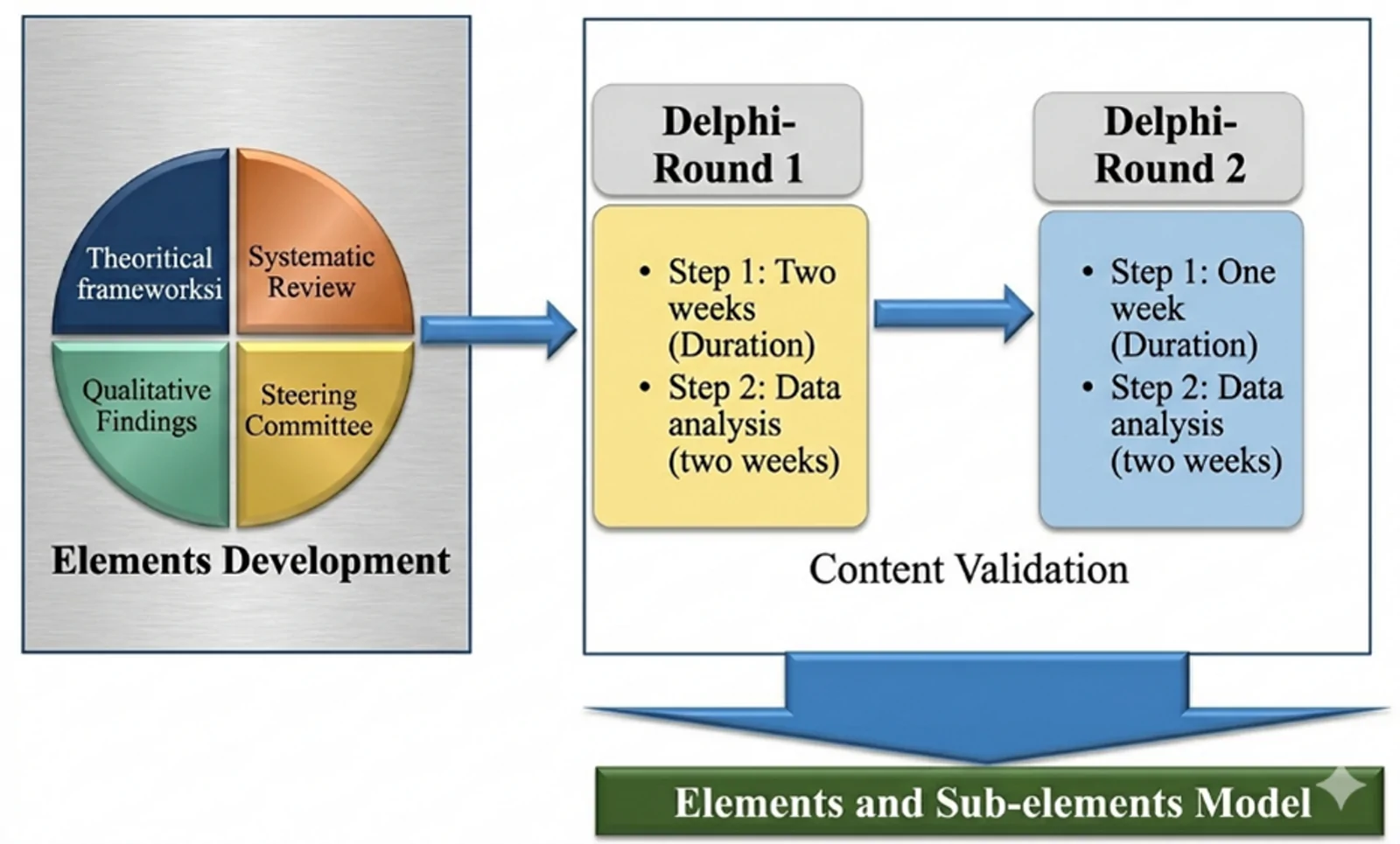
Procedures for the development and content validation of the IneRISE-IMAN model

Round 2 was conducted one week after panelists received individualized feedback summarizing group-level Round 1 results, including the I-CVI for each item and a synthesis of qualitative themes. The revised questionnaire was redistributed for re-evaluation over one week. This process aimed to consolidate consensus, resolve residual uncertainties, and confirm that each revised element accurately represented the intended learning model. The overall Delphi procedure is illustrated in Fig. 1.

### Data analysis

Content validity was assessed at both item and scale levels. The Item-level Content Validity Index (I-CVI) was calculated as the proportion of panelists rating an item as highly relevant (scores 7–9). Scale-level content validity was evaluated using two complementary indices: the Average method (S-CVI/Ave), with a value ≥ 0.90 indicating excellent content validity, and the Universal Agreement method (S-CVI/UA), which represents the proportion of items achieving I-CVI = 1.00 across all panelists [34].

To account for chance agreement among panelists, inter-rater agreement was examined using Fleiss’ Kappa coefficient. It is important to note that this analysis assessed the degree of agreement among raters regarding item relevance, rather than constituting a psychometric reliability assessment of the instrument itself; accordingly, the term *inter-rater agreement* is used throughout this paper in preference to reliability [38]. Kappa values were interpreted using established benchmarks: < 0.40, poor agreement; 0.40–0.59, moderate agreement; 0.60–0.74, good agreement; and ≥ 0.75, excellent agreement [38]. Consistent with recommendations for content validation studies, Kappa values were interpreted alongside theoretical relevance and expert judgment rather than as absolute thresholds [39].

Qualitative data from open-ended responses were compiled and thematically summarized by a trained research assistant. The research team reviewed all suggested revisions and reached consensus on item retention, modification, or deletion. Final decisions were confirmed by the steering committee.

## Results

### Characteristics of the expert panel

**Fig. 2:**
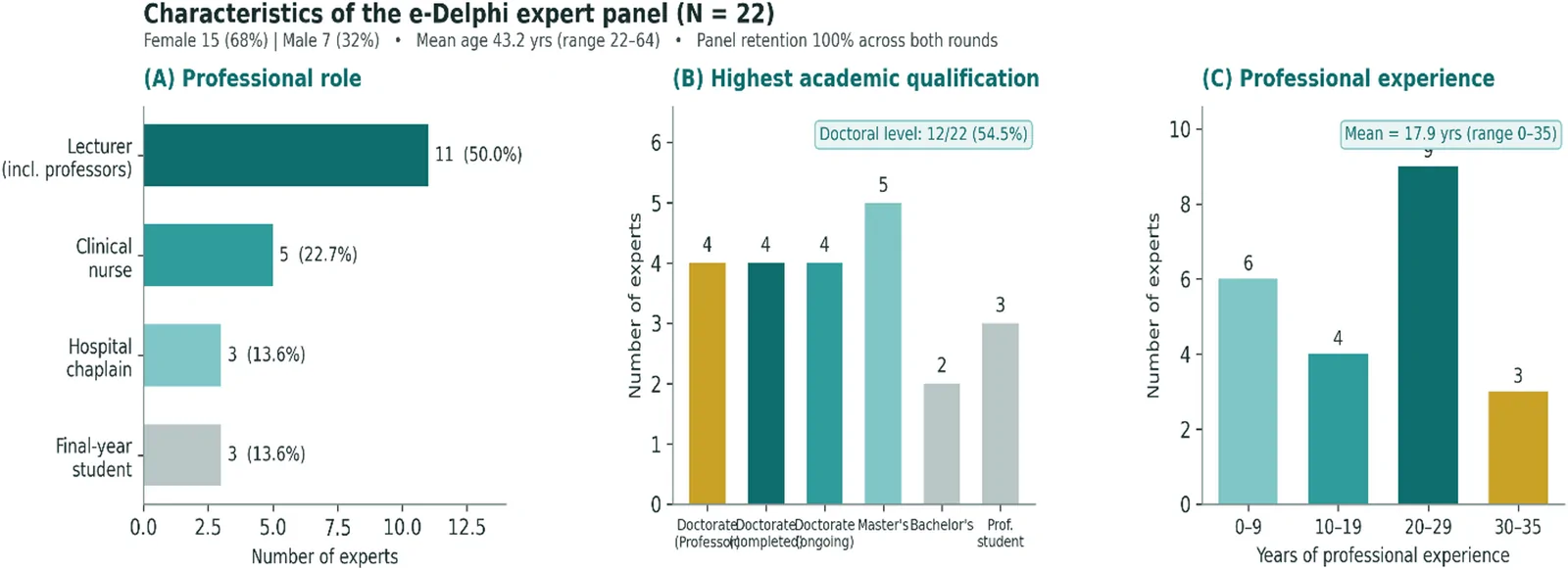
Characteristics of the e-Delphi expert panel (*N* = 22), showing (**A**) professional role, (**B**) highest academic qualification, and (**C**) years of professional experience

The Delphi panel comprised 22 participants representing participants’ professional roles, senior nursing students, academic qualifications, age distribution, work experience, gender, and institutional affiliations, demonstrating a diverse, highly qualified, and experienced expert panel with international representation. Figure 2 summarizes the characteristics of the Delphi panel (*N* = 22) involved in validating the Islamic Spiritual Care Learning Model.

The high proportion of doctoral-level experts (54.5%) and full professors (18.2%) strengthens the credibility of the consensus achieved, while the inclusion of chaplains and nursing students ensured that both theological and learner perspectives were represented in the validation process.

### Content validity and inter-rater agreement: round 1

The proposed IneRISE-IMAN framework demonstrated excellent content validity in the first Delphi round. Across the 71 items evaluated, the total I-CVI sum was 66.86, yielding an S-CVI/Ave of 0.94, exceeding the 0.90 threshold indicative of excellent scale-level content validity [39] Of the 71 items, 56 achieved universal agreement (I-CVI = 1.00), corresponding to an S-CVI/UA of 79%. These results indicate that most model elements were rated as highly relevant by all or nearly all panelists in the first round.

Inter-rater agreement analysis using Fleiss’ Kappa yielded a coefficient of 0.44, reflecting moderate agreement among panelists [38]. This level of agreement was considered acceptable and methodologically appropriate given two contextual factors. First, the panel’s deliberately interdisciplinary composition, spanning nursing educators, clinicians, chaplains, and students, introduces genuine diversity of perspective that is expected to produce moderate rather than perfect agreement in a first round and is indeed a strength rather than a limitation of the design. Second, content validation studies routinely demonstrate moderate inter-rater agreement at the first round, as the Delphi process is specifically designed to progressively consolidate consensus through iterative feedback [39]. The moderate Kappa therefore reflects meaningful expert engagement rather than superficial conformity. Full Round 1 content validity results are presented in Table 2.

**Table 2:** Round 1 content validation

NO	Element and sub elements model	Mean	Median	STD	I-CVI	UA
1	Lesson plan	8.41	9	0.89	0.95	1
2	**credit 1 credit (0**,**5 T**,** 0**,**5 P)**	**5.64**	**6**	**1.30**	**0.14**	**0**
3	*Course placement and format: Elective course 2 credit*	7.09	8	1.95	0.73	0
4	Supporting materials and readings based on Islamic teachings regarding spiritual care	8.41	8.5	0.65	1.00	1
5	Topic 1: Self-reflection: Who am I, what is my purpose in life? Why did I become a nurse? Deskripsi: proses berpikir secara mendalam dan kritis terhadap pengalaman atau tindakan mahasiswa selama ini	8.45	9	0.72	1.00	1
6	Topic 2: Spiritual concepts in nursing (general) Description: The general spiritual concept in nursing includes the definition, urgency, and role of nurses.	8.18	8	0.83	0.95	0
7	Topik 3: Spiritual (pillars of faith) and religious (pillars of Islam) concepts Description: Spiritual concepts in Islam include an understanding of the pillars of faith and Islam in the context of Islamic spiritual care.	7.91	8	0.95	0.95	0
8	Topic 4: The Concept of Humanity from Imam Al-Ghazali’s Perspective Description: The concept of physical, spiritual, and soul-nafs in the context of Islamic spiritual care.	8.32	9	0.82	1.00	1
9	Topic 5: The concept of human needs based on Maqashid Syariah Imam Asy-Syatibi Description: The need to maintain religion, reason, soul, descendants (social), and wealth in the context of Islamic spiritual care	8.32	8.5	0.76	1.00	1
10	Topic 6: Spiritual Needs of Muslim Patients Description: The spiritual needs of Muslim patients include their needs in terms of faith, morals, and sharia within the context of Islamic culture and spiritual care.	8.45	9	0.72	1.00	1
11	Topic 7: Islamic Spiritual Care Nursing Process Description: The nursing process includes spiritual assessment, establishing a spiritual nursing diagnosis, planning, implementation, and evaluation.	8.55	9	0.66	1.00	1
12	Topic 8: Fiqh of Worship and End-of-Life Care Description: Sharia laws regarding the implementation of purification, prayer, and end-of-life care in an Islamic manner.	8.36	8	0.71	0.95	0
13	Topic 9: Islamic Ethics in the Care of Muslim Patients Description: Moral, ethical, and cultural issues in the care of Muslim patients.	8.32	8	0.63	1.00	1
14	Topic 10: Evidence-Based Islamic Spiritual Care Interventions Description: Various types of Islamic spiritual care interventions include guidance on worship during illness, end-of-life support, and evidence-based Islamic complementary therapies.	8.59	9	0.58	1.00	1
15	**Topic 11: Islamic Guidance and Counseling Ethics** **Description: Islamic communication**,** guidance ethics**,** stages of Islamic guidance and counseling in the context of Islamic spiritual care.**	**7.45**	**8**	**1.41**	**0.59**	**0**
16	Topic 12: Case scenarios of Muslim patients with spiritual and/or religious needs Description: Narrative descriptions of spiritual and/or religious cases of Muslim patients for case study learning.	8.41	8.5	0.65	1.00	1
17	2. Student-centered learning method Description: An active, participatory, and experience-based learning process for students.	8.41	9	0.72	1.00	1
18	1. Flipped Classroom Description: Learning activities deliver material outside the classroom (via videos, readings, or online modules), while in-class time is used for discussions, exercises, problem-solving, and other interactive activities.	8.18	8	0.72	1.00	1
19	1. Case Discussion and Presentation Description: An active learning method that uses simulated cases as discussion material to encourage students to analyze situations, identify problems, formulate solutions, and make decisions based on theory, experience, and critical reasoning.	8.27	8	0.75	1.00	1
20	1. Practice of Islamic Spiritual Care Intervention Skills Description: Student psychomotor activities to meet the spiritual-religious needs of Muslim patients.	8.27	8	0.81	0.95	0
21	(1). Practice (guidance for purification, prayer, and supplication) Description: Helping patients fulfill their religious needs (sharia) during illness: tayamum / ablution, praying while sitting/lying down, and prayer guidance.	8.50	9	0.78	1.00	1
22	(2). Practice (guidance on purification, prayer, and supplication) Description: Helping patients select evidence-based Islamic complementary interventions to apply to their patients.	8.45	9	0.66	1.00	1
23	(3). End-of-Life Care Practice Description: Helping patients achieve a good end (talking), accompanying families during their time of grief (destiny and delivering bad news), principles of corpse care	8.55	9	0.58	1.00	1
24	(4). Active-empathetic and Islamic communication practices Description: Practice communicating in various situations (spiritual assessment communication, intervention communication, bad news communication)	8.41	9	0.83	0.95	0
25	(5). Simulation Description: A learning method that mimics real-world situations in a structured manner, allowing students to practice skills, decision-making, and communication in a safe environment, without risk to actual patients.	8.36	8.5	0.71	1.00	1
26	(6). Role play Description: Role play is an active learning method in which students play specific roles (e.g., nurses, patients, family members, or other healthcare professionals) in a scenario designed to represent a real or simulated situation.	8.36	8	0.57	1.00	1
27	(6). Reflection activities after each topic is completed Description: Reflection is the process of thinking deeply and critically about a particular experience or action to understand its meaning, evaluate its impact, and plan for future improvements. In an Islamic spiritual care context, reflection through *tafakur* and *muhasabah* enables students to contemplate learning experiences, evaluate themselves, and strengthen spiritually grounded nursing practice.	8.27	8.5	0.91	0.95	0
28	4. Learning evaluation Description: Assessment that covers cognitive, affective, psychomotor, and spiritual aspects.	8.36	8	0.57	1.00	1
29	(1). Written exam Description: a form of assessment that tests students’ cognitive understanding through written media, either in the form of an objective test (multiple choice) or an essay test.	8.05	8	0.77	1.00	1
30	(2). Practical exam Description: a form of assessment that assesses students’ psychomotor skills or actual performance in situations close to reality, such as clinical skills, communication, or nursing actions.	8.14	8	0.81	1.00	1
31	(3). Final reflection Description: A narrative or portfolio-based assessment that assesses students’ ability to reflect on learning or practical experiences, relate them to theory, and identify personal and professional learning.	8.18	8	0.78	1.00	1
32	B. Learning Facilities Description: Educational facilities include all physical facilities and infrastructure that support teaching and learning activities, such as classrooms, laboratories, libraries, learning aids, and information technology.	8.55	9	0.58	1.00	1
33	1. Classroom and Learning Laboratory Description: Lecture and practical facilities, such as simulation rooms for Islamic spiritual care practices.	8.55	9	0.50	1.00	1
34	2. Islamic Multimedia and E-modules Description: Availability of digital learning media, textbooks, and modules containing Islamic values	8.32	8.5	0.76	1.00	1
35	1. Qualifications of the teaching team (lecturers, practitioners, clergy) Description: Relevant academic background and teaching experience (minimum Master’s degree)	8.64	9	0.48	1.00	1
36	2. Islamic Pedagogical Competence Description: The ability to teach using an Islamic approach	8.36	9	0.83	0.95	0
37	3. Spiritual care experience Description: Experience in teaching, practicing, or researching Islamic spiritual care.	8.27	8	0.81	1.00	1
38	4. The teaching team has a role model attitude Description: The teaching team demonstrates exemplary morals and spirituality.	8.50	9	0.66	1.00	1
39	Student Characteristics	8.23	8	0.73	1.00	1
40	**(1). Must be in at least semester III.** **Description: Semester three refers to the second (initial) year in a higher education program (usually a bachelor’s/S1)**,** which usually takes place in the odd semester (around August–December).**	**7.00**	**7**	**1.65**	**0.55**	**0**
41	**(2). Age** **description: the number of years calculated from a person’s birth to a certain time (usually the time of data collection)**,** and is usually expressed in years.**	**6.36**	**6**	**1.49**	**0.41**	**0**
42	**(3). Gender** **description: refers to a person’s biological attributes**,** generally classified as male or female**	**6.00**	**6.5**	**1.45**	**0.45**	**0**
43	**(3). Religion** **description: a system of beliefs and spiritual practices followed by an individual or group**,** usually including belief in a God.**	**7.18**	**8**	**1.59**	**0.64**	**0**
44	2. Self-efficacy and resilience Description: Students’ confidence and resilience in their ability to provide spiritual care	8.32	8	0.70	1.00	1
45	*3. Learning Styles* *Description: Student learning preferences and strategies*	6.82	7	2.01	0.73	0
46	Peer support and discussion Description: Interaction and cooperation with classmates	8.36	9	0.77	1.00	1
47	Islamic-themed learning environment Description: A campus environment that supports Islamic values and practices.	8.18	8	0.72	1.00	1
48	3. Support for technological innovation in learning Description: Utilization of technology to support Islamic learning	8.14	8	0.81	1.00	1
49	1. Situation description Definition: Students’ ability to describe learning situations	8.36	9	0.88	1.00	1
50	2. Expression of feelings Description: Students express emotions or feelings related to their Islamic spiritual care learning experiences.	8.41	9	0.72	1.00	1
51	3. Self-evaluation Description: Ability to assess the strengths and weaknesses of actions taken	8.32	8	0.70	1.00	1
52	4. Analysis Description: The ability to understand the meaning of experiences and learning	8.32	8.5	0.76	1.00	1
53	5. Action Plan Description: Designing future actions based on the results of the reflection	8.59	9	0.58	1.00	1
54	6. Reflection Notes (Conclusion) Description: Compiling reflection notes and summarizing the reflection results for professional growth.	8.32	8.5	0.76	1.00	1
55	1. Understand the concept of Islamic spiritual care Description: Understanding the theory and principles of Islamic spiritual care	8.32	8.5	0.76	1.00	1
56	2. Applying the concept of the spiritual nursing process Description: Able to apply spiritual-based nursing care steps in case scenarios	8.41	9	0.72	1.00	1
57	3. Spiritual Beliefs and internalization Description: Evaluates one’s own faith values and their influence on Islamic spiritual care practices, as well as assesses the patient’s spiritual beliefs, which may differ from one’s own.	8.32	9	0.82	1.00	1
58	Competency - Islamic Communication Description: Students possess the ability to communicate actively and spiritually in an Islamic, empathetic, and appropriate manner.	8.50	9	0.58	1.00	1
59	1. Empathetic Description: Students have the ability to demonstrate concern and empathy when communicating.	8.45	9	0.66	1.00	1
60	2. Therapeutic Description: Students have the ability to communicate using therapeutic communication techniques that support healing.	8.55	9	0.58	1.00	1
61	2. Active Description: Students listen and respond actively to communication.	8.82	9	0.39	1.00	1
62	Psychomotor Competence Description: Students have the ability to perform real-life Islamic spiritual care actions on simulated patients.	8.32	8.5	0.76	1.00	1
63	1. Spiritual assessment Description: Students have the ability to assess patients’ spiritual needs.	8.50	9	0.78	1.00	1
64	2. Islamic Intervention Description: Students have the ability to provide nursing interventions using an Islamic approach.	8.36	8	0.64	1.00	1
65	3. Interprofessional Collaboration Description: Collaborating across professions in spiritual care	8.32	8.5	0.76	1.00	1
66	4. Documentation of care Description: Documenting Islamic spiritual care according to nursing standards.	8.41	8.5	0.65	1.00	1
67	Competency - Attitude Description: Students are able to demonstrate an empathetic and spiritually supportive presence.	8.59	9	0.49	1.00	1
68	1. Presence Description: Accompanying the patient completely, physically and spiritually	8.59	9	0.58	1.00	1
69	2. Cultural sensitivity Description: Aware of the diversity of patients’ spiritual-religious beliefs	8.45	9	0.72	1.00	1
70	3. Islamic Ethics Description: Students demonstrate morals and ethical values in practice.	8.55	9	0.66	1.00	1
71	4. Trust Description: Students are able to foster a sense of security and trust in patients.	8.64	9	0.57	1.00	1

### Refinement of model elements between rounds

While most items achieved consensus in Round 1, six sub-elements related to course placement and format, learning style, and student semester requirements did not meet the pre-defined I-CVI threshold of ≥ 0.78 and required revision prior to Round 2. Qualitative feedback from panelists provided substantive direction for these revisions, with three dominant themes emerging from the open-ended responses.

First, regarding learning plan flexibility, several panelists emphasized that Islamic spiritual care content should be embedded in local institutional curricula rather than offered as a fixed, standalone course, enabling study programs to integrate content across multiple courses to align with their specific learning outcomes and institutional profiles. Second, regarding student requirements, a consensus emerged that placement of Islamic spiritual care education in semester III is premature; panelists recommended a minimum of semester V, ensuring students have first established foundational nursing knowledge and general spiritual care concepts before engaging with the more complex Islamic contextualization. Additionally, multiple panelists noted that religious affiliation should not constitute an absolute prerequisite for participation, as non-Muslim students may equally benefit from intercultural spiritual care competence. Third, regarding learning style, panelists clarified that it should not serve as a fixed student entry criterion but rather as a variable that informs pedagogical adaptation. Learning methods should be responsive to diverse student learning preferences rather than prescriptive. Representative expert quotes supporting these themes are presented in Table 3.

**Table 3:** Important recommendations from open-ended questions in the first round

No	Theme	Sub theme	coding	Quotes	Code
1	Learning plans	flexibility of learning plans	local content curriculum	Local content curriculum is a curriculum determined by an institution that can be made unique	P11
			needs and profile of educational institutions	The embodiment in the form of local content returns to the needs and learning outcomes, as well as the profile built by educational institutions.	P12
			includes several courses	For the Islamic spiritual care curriculum, it can be included in several courses, depending on the study program’s strengths, so that the total can exceed 1 credit unit.	P22
2	Topics/material	Module	learning topic, module	Learning topics are included in the learning module	P5, P7, P9, P12
3	Learning methods	Reflective-flipped classroom methods	students centre learning, flipped classroom	Student-centered learning (SCL) and flipped classroom methods are highly suitable for spiritual care education because they emphasize active involvement, contextual understanding, and personal reflection, helping students integrate Islamic values into real nursing practice and encouraging the development of health workers who are clinically competent and spiritually informed.	P10
			Reflection, debriefing	Reflection can be a valuable method, but if it’s applied to every topic, it can become a mere ritual. Perhaps it could be combined with debriefing.	P22
4	Learning evaluation	Comprehensive evaluation	written test, practical test	The evaluation method must be holistic. contextual, and reflective, not only based on written tests, but also involving observation, real practice, and assessment of attitudes and reflection.	P11
			narrative reflection	Both narrative reflection and portfolio assessments are highly relevant in assessing a person’s understanding of spirituality, although they pose challenges due to their inherent subjective nature. A possible suggestion for this assessment is the creation of a scoring rubric to minimize bias and objectify each answer.	P12
5	Student Characteristics	student requirements	at least in semester V	Semester III is too early for Islamic spiritual care; students must first get basic nursing (general spiritual), at least in semester V	P10, P21
				Religion is not an absolute requirement for studying Islamic spiritual care.	P6, P7,P11
6	Learning style	learning style	learning method	match with learning methods and objectives	P10
				not an absolute requirement but the learning method can be adjusted	P14
7	Lecturers have pedagogical competence and qualification	lecturer requirements	detalied definition	need a more detailed definition regarding this competency	P22
8	Lecturers have role models		role modeling	The basic principle of the Islamic spiritual education approach is role modelling.	P14
9	Lecturers have exeperience		experience	It is necessary to enrich insight with real experiences that have been experienced	P11
10	Technology-based interactive modules	e-module interactive	digital modules	The availability of digital modules is a medium that facilitates the interactive learning process.	P10
11	Gibbs reflection cycle	Reflection process	accelerate understanding	It is crucial to accelerate understanding and facilitate the learning process effectively with the latest references.	P10
			emotion-feelings	Spirituality is closely related to emotions and feelings	P14
12	Attitude	cultural sensitivity	spiritual-religion diversity	Spiritual and religious diversity has a high sensitivity in social life and requires caution in life interactions, including those in hospitals.	P7, P12, P15
13	Communication	active communication	balance in listening and providing	Active doesn’t mean dominant. Students are expected to balance listening and providing professional opinions.	P10

Based on this qualitative synthesis, the identified sub-elements were substantially revised. Specifically, the course placement item was reframed to emphasize curricular flexibility rather than a fixed credit structure; the student semester requirement was adjusted to semester V as the recommended minimum; religious affiliation was removed as a mandatory criterion; and the learning style item was reconceptualized as a pedagogical consideration rather than a student selection criterion.

### Content Validity and inter-rater agreement: round 2

Following targeted revision, the two previously non-consensus items were re-evaluated in Round 2. Both items achieved full consensus: course placement and format attained an I-CVI of 1.00 (mean = 8.73, SD = 0.54), and learning style similarly achieved an I-CVI of 1.00 (mean = 8.32, SD = 1.06), as presented in Table 4. Full panel retention was maintained across both rounds, with all 22 panelists completing Round 2 (response rate: 100%). The achievement of S-CVI/UA = 1.00 in Round 2 confirms that complete universal agreement was reached across all model elements following the iterative revision process.

**Table 4:** Second round content validation

sub-elements	mean	median	SD	I-CVI	UA
course placement, and format	8.73	9	0.54	1	1
learning style	8.32	9	1.06	1	1

### Core structure of the final learning model

The final learning model consists of seven interconnected core elements: curriculum, students, lecturers, learning environment, learning facilities, reflective learning processes, and spiritual care competencies in the Islamic context. Rather than operating as isolated components, these elements interact dynamically through a central reflective learning process.

**Fig. 3:**
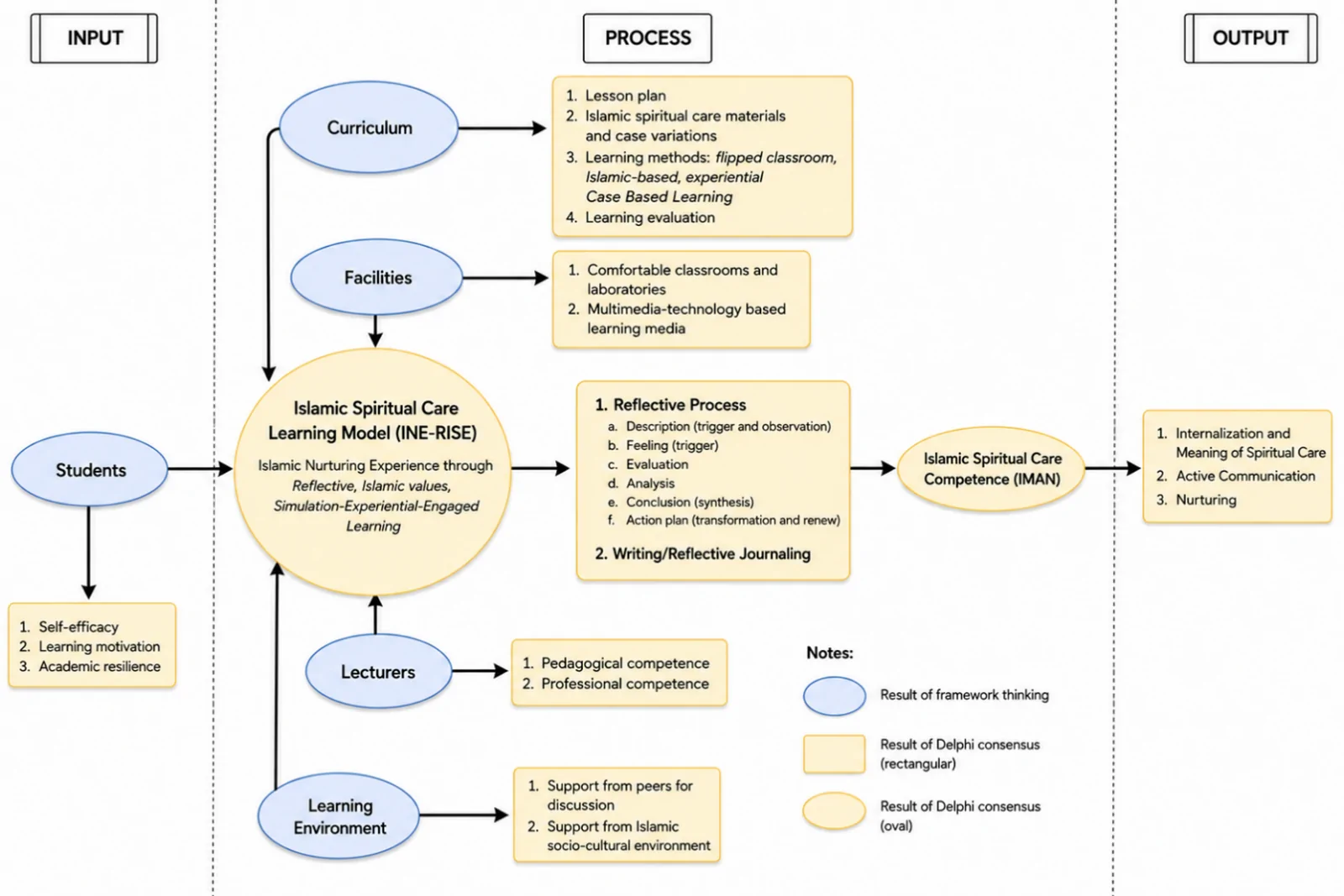
IneRISE IMAN model result from Delphi

Expert consensus consistently emphasizes reflection as the pivotal mechanism that connects educational inputs to competency outcomes. Reflection is conceptualized as a structured process that includes description, emotional awareness, evaluation, analysis, conclusion, and action planning [40]. This process is deemed essential for facilitating the internalization of Islamic values and converting theoretical knowledge into spiritually aligned nursing practice. The model Reflective, as the core of the spiritual care learning process in the Islamic context, can be called IneRISE-IMAN, see Fig. 3.

Figure 3 shows the final validated IneRISE-IMAN model, organized into three interacting domains: Input, Process, and Output, grounded in three complementary theoretical foundations: Al-Ghazali’s Islamic theory of human nature (ontological), constructivist learning theory (epistemological), and McCormack and McCance’s Person-Centered Nursing Framework (praxis). The Input domain includes four elements confirmed through expert consensus: (1) Curriculum, which includes lesson plans, Islamic spiritual care materials, active learning methods (such as flipped classroom, Islamic-based, experiential Case Base Learning/CBL), and learning evaluation; (2) Facilities, such as classrooms, laboratories, and multimedia learning tools; (3) Lecturers, whose pedagogical and professional skills are essential for effective spiritual care education; and (4) Students, whose self-efficacy, motivation for learning, and academic resilience were identified in Round 1 as key entry variables, reflecting Vygotsky’s Zone of Proximal Development and Piaget’s schema adaptation. The Process domain is centered around the INE-RISE mechanism, which integrates all inputs toward competency outcomes through two validated learning activities: (1) a six-stage Reflective Process description, feeling, evaluation, analysis, conclusion, and action planning based on Gibbs’ reflective cycles and enriched by Islamic practices; and (2) Reflective Journaling. Panelists confirmed in Round 2 (I-CVI = 1.00) that reflection must be rooted in an Islamic epistemological orientation. The learning environment, including peer support and Islamic socio-cultural context, was acknowledged as a crucial enabling condition. The Output domain, mediated through Islamic Spiritual Care Competence (IMAN), defines three outcomes with full agreement (S-CVI/UA = 1.00): Internalization and Meaning of Spiritual Care, Active Communication, and Nurturing. These outcomes align with Person-Centered Nursing standards while integrating Islamic values of tawakkul, sabr, and raja’ into compassionate nursing practice.

## Discussion

This study focused on developing and validating a spiritual care learning framework for undergraduate nursing in the Islamic context, integrating constructivist pedagogy, person-centered care, and Islamic spiritual principles. The findings demonstrate that the IneRISE-IMAN model is both logically coherent and contextually relevant, offering a structured approach to addressing existing gaps in spiritual care learning in Muslim-majority settings. The validated model advances a conceptually grounded, culturally responsive, and pedagogically coherent alternative to existing frameworks that have predominantly emerged from Western or secular educational traditions [9, 13, 41, 42]. The following discussion situates the IneRISE-IMAN model within the broader literature on spiritual care education, reflective pedagogy, person-centered nursing, and Islamic educational philosophy, highlighting both its distinctive contributions and its alignment with international nursing education standards.

### Integration of theoretical frameworks within IneRISE-IMAN

The IneRISE-IMAN model operationalizes constructivist learning theory by placing reflective learning at the core of students’ active construction of meaning from their educational experiences. Constructivism emphasizes learning as an active, experiential, and reflective process rather than merely the transmission of knowledge [24, 28]. In this study, expert consensus strongly emphasized the importance of structured reflection, including description, emotional awareness, evaluation, analysis, conclusion, and action planning, as essential for linking educational inputs to spiritual care competencies. This finding aligns with previous research highlighting reflection as a key strategy for developing self-awareness, ethical sensitivity, and caring behaviors among nursing students [26, 40, 43, 44].

Importantly, the IneRISE-IMAN model conceptually extends conventional reflective learning by situating reflection within an Islamic epistemological framework. Concepts such as *tafakkur* (deep reflection), *niyyah* (moral intentionality), and *tazkiyatun nafs* (self-purification) were identified by expert panelists as essential dimensions that enrich the reflective process in Islamic spiritual care education [22, 45]. This conceptual alignment resonates with Al-Ghazali’s perspective on human development, which emphasizes the integration of knowledge, moral character, and spiritual awareness as inseparable dimensions of genuine learning [46].

It is important to acknowledge that the current study did not evaluate the implementation or educational impact of these reflective processes. The integration identified reflects expert consensus on curricular relevance rather than empirical evidence of effectiveness. Therefore, future implementation and evaluation studies are necessary to investigate how reflective practices within the IneRISE-IMAN framework affect students’ learning experiences, professional identity formation, and competencies in spiritual care. Comparative analysis with analogous frameworks internationally reveals both shared principles and distinctive contributions of IneRISE-IMAN. Both the Attard et al. (2019) spiritual care competency framework and the wider EPICC (Enhancing Nurses’ and Midwives’ Competence in Providing Spiritual Care through Innovative Education and Compassionate Care) project, of which it forms part, used a modified Delphi process to reach expert consensus and identified reflective practice, self-awareness, and respectful, person-centred communication as core competency domains [17]. Importantly, the EPICC outputs do not disregard context: the framework and its subsequent Gold Standard Education Matrix explicitly address the cultural, social, and political environment in which spiritual care competency develops, and the EPICC standard was deliberately designed to be adapted, culturally and linguistically, across diverse European settings. What they do not set out to provide, however, is the epistemological and theological grounding of a specific faith tradition; developed within religiously pluralistic and predominantly post-Christian European contexts, they do not articulate the Islamic philosophical anthropology or faith-specific practices that shape spiritual care in Muslim-majority settings. The Ross et al. (2022) EPICC education matrix [18], for example, maps the factors that facilitate or hinder competency development, including educator attitudes and organisational support, yet its content remains generic with respect to religious tradition rather than tailored to Islamic spiritual care. The IneRISE-IMAN model therefore extends this established body of work by embedding Islamic moral philosophy, faith-specific competencies, and culturally informed pedagogical strategies within a single content-validated educational framework, complementing rather than replacing the generic EPICC standard.

### Alignment with person-centered nursing principles

The validated model aligns closely with Person-Centered Nursing (PCN), which emphasizes therapeutic relationships, respect for patients’ values, and holistic engagement with individuals and families [3, 29]. Expert consensus indicates that competence in Islamic spiritual care extends beyond mere technical interventions; it necessitates empathetic presence, ethical sensitivity, and culturally congruent communication aspects that closely align with PCN principles and reinforce the inherently relational and context-dependent nature of spiritual care.

Within the IneRISE-IMAN framework, PCN is operationalized through reflective engagement with patients’ spiritual narratives and moral concerns, particularly during illness, suffering, and end-of-life care. By incorporating Islamic beliefs commonly held by Muslim patients, including trust in God (*tawakkul*), patience (*sabr*), and hope (*raja’*), the model promotes spiritually congruent care while embracing universal humanistic values [11, 47]. This contextualization directly addresses concerns raised in previous studies suggesting that generic spiritual care learning may inadequately equip nurses to meet the faith-informed needs of Muslim patients [12, 16].

### Central role of educators, learning environment, curriculum design, and educational facilities

Consistent with both constructivist and Islamic teaching traditions, the Delphi findings underscored the critical role of lecturers as both pedagogical facilitators and moral and spiritual role models. Expert consensus suggests that educators’ lived clinical experience, ethical conduct, and spiritual maturity significantly influence students’ capacity to engage in reflective, spiritually sensitive practice, a finding aligned with evidence that spiritual care competencies are shaped not only by curricular content but also by educators’ attitudes and modeling behaviors [7, 18, 43].

From an Islamic educational perspective, this emphasis reflects the principle of *uswah hasanah* (exemplary role modeling), wherein values are transmitted through observable conduct rather than instruction alone [31, 48]. IneRISE-IMAN explicitly incorporates this principle by positioning lecturers as integral inputs within the learning system, reinforcing the moral and spiritual dimensions of nursing education. The learning environment was similarly recognized as an enabling condition for effective spiritual care education. Experts emphasized the importance of psychologically safe spaces, peer support, and Islamic socio-cultural nuances that foster openness during reflective and experiential learning activities [12, 43].

The curriculum is equally central to developing reflective competencies. Recent evidence demonstrates that *flipped classrooms* and student-centered approaches significantly enhance critical thinking, engagement, and self-directed learning among nursing students [49–51]. The integration of structured lesson planning, active learning strategies, and formative evaluation creates systematic opportunities for reflection and clinical reasoning, enabling deeper cognitive and spiritual engagement with Islamic spiritual care content [52].

Student characteristics, particularly self-efficacy, academic resilience, and learning motivation, were identified by panelists as foundational entry variables that directly influence learning outcomes [53, 54]. Research consistently demonstrates that treating nursing students as active participants rather than passive recipients enhances metacognitive development, professional identity formation, and preparedness for clinical practice. Finally, educational facilities, including simulation laboratories, multimedia-based learning media, and interactive learning environments, were affirmed as essential supports that connect theoretical knowledge with experiential practice. Structured debriefing following simulation activities has been specifically identified as a mechanism for deepening clinical judgment and integrating knowledge with values-based spiritual care [55, 56].

### Curricular adaptation and implementation

The IneRISE-IMAN model was developed through a systematic three-phase research program involving needs assessment, content validation, and planned feasibility evaluation. It mirrors the evidence-based curriculum development framework for nursing education advocated by Iwasiw et al. (2020) [57], which moves from contextual scanning and philosophical foundations to curriculum design, implementation, and ongoing evaluation. This alignment ensures the model is not just a conceptual construct but a practically implementable educational system with a replicable developmental trajectory suitable for undergraduate nursing programs.

Expert consensus confirmed that curriculum adaptation should favor institutional flexibility over strict, prescriptive frameworks. Panelists consistently emphasized that Islamic spiritual care content should be integrated into local institutional curricula, enabling nursing programs to incorporate model elements into existing courses, such as basic nursing, medical-surgical, palliative care, or community nursing, rather than requiring a separate, mandatory course. The suggested minimum placement in semester V, after students gain foundational nursing knowledge, ensures age-appropriate development and aligns with a spiral curriculum approach, where spiritual care skills are progressively deepened as students advance through clinical training [58].

In contrast to fragmented, single-intervention approaches wherein spiritual care is typically addressed through isolated workshops, one-time simulations, or single-semester electives [13, 43], IneRISE-IMAN provides a coherent, multi-element educational system in which curriculum delivery, lecturer role modeling, student characteristics, learning environment, facilities, reflective processes, and competency outcomes are explicitly interconnected. This systemic approach directly addresses the curricular fragmentation and absence of theoretical grounding identified as persistent barriers to effective spiritual care education in Muslim-majority nursing programs [12, 16], and aligns with international calls for structured, longitudinally embedded spiritual care education standards in undergraduate nursing [18].

### Advancement beyond existing spiritual care learning models

In contrast to spiritual care initiatives that rely on isolated interventions or short-term outcomes, IneRISE-IMAN offers a coherent, integrated learning model rooted in both educational theory and Islamic moral philosophy. By linking inputs, reflective processes, and competency outcomes, it addresses the curricular fragmentation and weak theoretical grounding previously identified in spiritual care education [13, 41].

The validated competencies span knowledge, attitudes, communication, and psychomotor skills, reflecting a holistic orientation consistent with contemporary calls for meaning-centered, person-centered nursing practice [11, 12, 21]. A defining feature of the model is its conceptualization of competence as the outcome of internalization rather than skill acquisition alone, which distinguishes it from primarily technical or cognitive training approaches. Viewed against established competency-based models, IneRISE-IMAN is best understood as building on, rather than departing from, the international evidence base. Consensus-based standards, such as the EPICC spiritual care education standard developed by van Leeuwen et al. (2021), articulate generic spiritual care competencies applicable across religious traditions [59], while scoping and integrative reviews of spiritual care pedagogy, including Rykkje et al. (2022), identify simulation, reflective journaling, and case-based learning as effective strategies [13] that are, however, typically applied without cultural or religious contextualization. IneRISE-IMAN draws these same evidence-based strategies together and situates them within an explicitly Islamic epistemological and theological framework. Its contribution therefore, lies less in introducing new pedagogical techniques than in the coherence of its pedagogical logic, which connects Islamic moral philosophy, constructivist learning theory, and Person-Centered Nursing outcomes within a single content-validated educational system.

### Implications for nursing education and future research

The findings indicate that IneRISE-IMAN has the potential to enhance curriculum alignment and ensure pedagogical coherence in undergraduate nursing programs situated in Muslim-majority contexts. By incorporating reflective learning, educator role modeling, and culturally relevant spiritual content, this model offers a versatile framework for faith-based spiritual care education. In practice, nurse educators and curriculum developers may use the validated IneRISE-IMAN framework as a structured guide for integrating Islamic spiritual care content into existing undergraduate nursing programs, whether as a standalone module or embedded across multiple courses in accordance with institutional learning outcomes. Healthcare institutions may similarly draw on the IMAN competency outcomes to inform clinical orientation programs for newly qualified nurses working in Muslim-majority patient populations. At the policy level, the model offers a theoretically and empirically grounded reference point for national nursing bodies and accreditation agencies seeking to articulate culturally responsive spiritual care competency standards [6, 19].

Future research should focus on establishing the construct validity of the IneRISE-IMAN model, assessing its feasibility and acceptability across diverse institutional settings, and evaluating its educational outcomes, including students’ spiritual care competencies, professional identity development, and clinical practice behaviors. Longitudinal and quasi-experimental designs would be particularly valuable for examining the model’s impact on students’ clinical practice experiences and patient care quality in Muslim-majority hospital settings.

### Strengths and limitations

A key strength of this study is the systematic development and content validation of the IneRISE-IMAN model through a rigorous two-round e-Delphi process involving experts in nursing education, clinical practice, and Islamic spiritual care. Achieving complete universal agreement (S-CVI/UA = 1.00) in Round 2, along with a high S-CVI/Ave of 0.94 in Round 1, provides strong evidence of the model’s content validity and positions it as a credible, consensus-driven educational framework. The clear theoretical grounding across three complementary traditions, Islamic philosophy, constructivist pedagogy, and person-centered nursing, further sets this model apart from less integrated initiatives in the existing literature. The interdisciplinary panel, composed of nursing lecturers, clinical nurses, hospital chaplains, and final-year nursing students, enhanced both the scholarly credibility and ecological validity of the validated framework.

However, several limitations must be acknowledged. First, the study focused solely on content validity and expert consensus; therefore, the findings do not provide evidence regarding the feasibility, acceptability, or effectiveness of the IneRISE-IMAN model in actual educational or clinical settings. Second, although the panel included participants from diverse professional backgrounds, the study was conducted within a specific cultural and religious context in Indonesia, which may limit the generalizability of the findings to highly pluralistic or non-Muslim-majority settings. Third, as a content validation study, it does not address how individual student factors, such as prior religious knowledge or intercultural competence, may influence the model’s educational impact.

Despite these limitations, the study offers a theoretically grounded and consensus-based foundation for future curriculum development and empirical research. Further studies are recommended to pilot the IneRISE-IMAN model within undergraduate nursing programs, assessing its feasibility, educational outcomes, and long-term clinical effects across different institutional contexts.

## Conclusion

The IneRISE-IMAN model, grounded in al-Ghazali’s Islamic philosophical anthropology, constructivist learning theory, and Person-Centered Nursing, demonstrates strong content validity as the first validated pedagogical framework for Islamic spiritual care education in undergraduate nursing. Achieving S-CVI/Ave = 0.94 in Round 1 and full universal agreement (S-CVI/UA = 1.00) in Round 2, the model establishes expert consensus across seven interacting educational elements that converge through structured reflective learning toward four competency outcomes: Internalization, Meaningful caring attitude, Active communication, and Nurturing Islamic interventions. Its primary novelty lies in conceptualizing spiritual care competence as internalization rather than skill acquisition and in systematically embedding Islamic moral philosophies: tafakkur, uswah hasanah, maqashid al-sharia within a constructivist pedagogical framework, distinguishing it from Western-based models that lack cultural and theological contextualization. For nursing education, the model offers curriculum developers a validated, structured guide for integrating Islamic spiritual care, whether as a standalone module or integrated across courses, prioritizing reflective pedagogy, lecturer role modeling, and simulation-based learning. For clinical practice, the IMAN competency outcomes provide measurable standards for orienting newly qualified nurses in Muslim-majority healthcare settings. Future research should establish construct validity, assess implementation feasibility across diverse institutions, and evaluate long-term impacts on students’ competencies, professional identity, and patient care quality.

## Supplementary Information

Supplementary Material 1.

## Data Availability

The datasets generated and/or analyzed during the current study are available from the corresponding author on reasonable request.

## Abbreviations

RISE: Reflective Islamic Spiritual Care Education
IMAN: Internalization, Meaning, Active, and Nurturing
I-CVI: Item-Level Content Validity Index
S-CVI/Ave: Scale-Level Content Validity Index, Average
S-CVI/UA: Scale-Level Content Validity Index, Universal Agreement
