# Supplementary material for: Development and validation of the IneRISE-Iman model: a spiritual care learning framework for undergraduate nursing students
Source: BMC Med Educ. 2026 Aug 5;26:1766. doi: 10.1186/s12909-026-10079-z (PMC13729435; doi:10.1186/s12909-026-10079-z)
Supplement: Supplementary file 1 [file 12909_2026_10079_MOESM1_ESM.pdf]

Supplementary files

First Round Delphi Questionnaire

Instructions for Completing the Delphi Questionnaire

Activity Objective

This questionnaire aims to establish consensus on the elements of the Islamic Spiritual Care learning model for undergraduate nursing students, based on the perspectives of experts, nursing practitioners, and professional students through two Delphi rounds.

Instructions

Participants are requested to evaluate each sub-element presented in this questionnaire according to its level of relevance and importance.

Please use a 9-point Likert scale (1–9) with the following criteria:

- **Scores 1–3**: Irrelevant / Not important
- **Scores 4–6**: Less relevant / Uncertain
- **Scores 7–9**: Highly relevant / Very important

Participants are encouraged to provide their ratings objectively, based on their knowledge, expertise, and professional experience.

Notes

- There are no right or wrong answers.
- Your responses are highly valuable in achieving group consensus.
- All responses will be kept confidential and used solely for research purposes.

NO	Element and sub elements model	1-3	4-9	7-9	comments
1	Lesson plan				
2	credit 1 credit (0,5 T, 0,5 P)				
3	*Course placement and format: Elective course 2 credit*				
4	Supporting materials and readings based on Islamic teachings regarding spiritual care				
5	Topic 1: Self-reflection: Who am I, what is my purpose in life? Why did I become a nurse?<br>Description: the process of thinking deeply and critically about students' experiences or actions so far				
6	Topic 2: Spiritual concepts in nursing (general)<br>Description: The general spiritual concept in nursing includes the definition, urgency, and role of nurses.				

NO	Element and sub elements model	1-3	4-9	7-9	comments
7	Topic 3: Spiritual (pillars of faith) and religious (pillars of Islam) concepts<br>Description: Spiritual concepts in Islam include an understanding of the pillars of faith and Islam in the context of Islamic spiritual care.				
8	Topic 4: The Concept of Humanity from Imam Al-Ghazali's Perspective<br>Description: The concept of physical, spiritual, and soul-nafs in the context of Islamic spiritual care.				
9	Topic 5: The concept of human needs based on Maqashid Syariah Imam Asy-Syatibi<br>Description: The need to maintain religion, reason, soul, descendants (social), and wealth in the context of Islamic spiritual care				
10	Topic 6: Spiritual Needs of Muslim Patients<br>Description: The spiritual needs of Muslim patients include their needs in terms of faith, morals, and sharia within the context of Islamic culture and spiritual care.				
11	Topic 7: Islamic Spiritual Care Nursing Process<br>Description: The nursing process includes spiritual assessment, establishing a spiritual nursing diagnosis, planning, implementing, and evaluating.				
12	Topic 8: Fiqh of Worship and End-of-Life Care<br>Description: Sharia laws regarding the implementation of purification, prayer, and end-of-life care in an Islamic manner.				
13	Topic 9: Islamic Ethics in the Care of Muslim Patients<br>Description: Moral, ethical, and cultural issues in the care of Muslim patients.				
14	Topic 10: Evidence-Based Islamic Spiritual Care Interventions<br>Description: Various types of Islamic spiritual care interventions include guidance on worship during illness, end-of-life support, and evidence-based Islamic complementary therapies.				
15	Topic 11: Islamic Guidance and Counseling EthicsDescription: Islamic communication, guidance ethics, stages of Islamic guidance and counseling in the context of Islamic spiritual care.				

NO	Element and sub elements model	1-3	4-9	7-9	comments
16	Topic 12: Case scenarios of Muslim patients with spiritual and/or religious needs<br>Description: Narrative descriptions of spiritual and/or religious cases of Muslim patients for case study learning.				
17	2. Student-centered learning method<br>Description: An active, participatory, and experience-based learning process for students.				
18	Flipped Classroom<br>Description: Learning activities deliver material outside the classroom (via videos, readings, or online modules), while in-class time is used for discussions, exercises, problem-solving, and other interactive activities.				
19	Case Discussion and Presentation<br>Description: An active learning method that uses simulated cases as discussion material to encourage students to analyze situations, identify problems, formulate solutions, and make decisions based on theory, experience, and critical reasoning.				
20	Practice of Islamic Spiritual Care Intervention Skills<br>Description: Student psychomotor activities to meet the spiritual-religious needs of Muslim patients.				
21	(1). Practice (guidance for purification, prayer, and supplication)<br>Description: Helping patients fulfill their religious needs (sharia) during illness: tayamaum, praying while sitting/lying down, and prayer guidance.				
22	(2). Practice (guidance on purification, prayer, and supplication)<br>Description: Helping patients select evidence-based Islamic complementary interventions to apply to their patients.				
23	(3). End-of-Life Care Practice<br>Description: Helping patients achieve a good end (talking), accompanying families during their time of grief (destiny and delivering bad news), principles of corpse care				
24	(4). Active-empathetic and Islamic communication practices<br>Description: Practice communicating in various situations (spiritual assessment				

NO	Element and sub elements model	1-3	4-9	7-9	comments
	communication, intervention communication, bad news communication)				
25	(5). Simulation<br>Description: A learning method that mimics real-world situations in a structured manner, allowing students to practice skills, decision-making, and communication in a safe environment, without risk to actual patients.				
26	(6). Role play<br>Description: Role play is an active learning method in which students play specific roles (e.g., nurses, patients, family members, or other healthcare professionals) in a scenario designed to represent a real or simulated situation.				
27	(6). Reflection activities after each topic is completed<br>Description: Reflection is the process of thinking deeply and critically about a particular experience or action to understand its meaning, evaluate its impact, and plan for future improvements. In an educational context, reflection allows students to connect learning experiences with their knowledge, values, and professional development.				
28	4. Learning evaluation<br>Description: Assessment that covers cognitive, affective, psychomotor, and spiritual aspects.				
29	(1). Written exam<br>Description: a form of assessment that tests students' cognitive understanding through written media, either in the form of an objective test (multiple choice) or an essay test.				
30	(2). Practical exam<br>Description: a form of assessment that assesses students' psychomotor skills or actual performance in situations close to reality, such as clinical skills, communication, or nursing actions.				
31	(3). Final reflection<br>Description: A narrative or portfolio-based assessment that assesses students' ability to reflect on learning or practical experiences, relate them to theory, and identify personal and professional learning.				

NO	Element and sub elements model	1-3	4-9	7-9	comments
32	B. Learning FacilitiesDescription: Educational facilities include all physical facilities and infrastructure that support teaching and learning activities, such as classrooms, laboratories, libraries, learning aids, and information technology.				
33	1. Classroom and Learning Laboratory Description: Lecture and practical facilities, such as simulation rooms for Islamic spiritual care practices.				
34	2. Islamic Multimedia and E-modules Description: Availability of digital learning media, textbooks, and modules containing Islamic values				
35	1. Qualifications of the teaching team (lecturers, practitioners, clergy)<br>Description: Relevant academic background and teaching experience (minimum Master's degree)				
36	2. Islamic Pedagogical Competence Description: The ability to teach using an Islamic approach				
37	3. Spiritual care experience<br>Description: Experience in teaching, practicing, or researching Islamic spiritual care.				
38	4. The teaching team has a role model attitude Description: The teaching team demonstrates exemplary morals and spirituality.				
39	Student Characteristics				
40	(1). Must be in at least semester III.Description: Semester three refers to the second (initial) year in a higher education program (usually a bachelor's/S1), which usually takes place in the odd semester (around August–December).				
41	(2). Age<br>description: the number of years calculated from a person's birth to a certain time (usually the time of data collection), and is usually expressed in years.				
42	(3). Gender<br>description: refers to a person's biological attributes, generally classified as male or female				
43	(3). Religion<br>description: a system of beliefs and spiritual				

NO	Element and sub elements model	1-3	4-9	7-9	comments
	practices followed by an individual or group, usually including belief in a God.				
44	2. Self-efficacy and resilience<br>Description: Students' confidence and resilience in their ability to provide spiritual care				
45	*3. Learning Styles*<br>*Description: Student learning preferences and strategies*				
46	Peer support and discussion<br>Description: Interaction and cooperation with classmates				
47	Islamic-themed learning environment<br>Description: A campus environment that supports Islamic values and practices.				
48	3. Support for technological innovation in learning<br>Description: Utilization of technology to support Islamic learning				
49	1. Situation description<br>Definition: Students' ability to describe learning situations				
50	2. Expression of feelings<br>Description: Students express emotions or feelings related to their Islamic spiritual care learning experiences.				
51	3. Self-evaluation<br>Description: Ability to assess the strengths and weaknesses of actions taken				
52	4. Analysis<br>Description: The ability to understand the meaning of experiences and learning				
53	5. Action Plan<br>Description: Designing future actions based on the results of the reflection				
54	6. Reflection Notes (Conclusion)<br>Description: Compiling reflection notes and summarizing the reflection results for professional growth.				
55	1. Understand the concept of Islamic spiritual care<br>Description: Understanding the theory and principles of Islamic spiritual care				
56	2. Applying the concept of the spiritual nursing process<br>Description: Able to apply spiritual-based nursing care steps in case scenarios				

NO	Element and sub elements model	1-3	4-9	7-9	comments
57	3. Spiritual Beliefs and internalization<br>Description: Evaluates one's own faith values and their influence on Islamic spiritual care practices, as well as assesses the patient's spiritual beliefs, which may differ from one's own.				
58	Competency - Islamic Communication<br>Description: Students possess the ability to communicate actively and spiritually in an Islamic, empathetic, and appropriate manner.				
59	1. Empathetic<br>Description: Students have the ability to demonstrate concern and empathy when communicating.				
60	2. Therapeutic<br>Description: Students have the ability to communicate using therapeutic communication techniques that support healing.				
61	2. ActiveDescription: Students listen and respond actively to communication.				
62	Psychomotor Competence<br>Description: Students have the ability to perform real-life Islamic spiritual care actions on simulated patients.				
63	1. Spiritual assessment<br>Description: Students have the ability to assess patients' spiritual needs.				
64	2. Islamic Intervention<br>Description: Students have the ability to provide nursing interventions using an Islamic approach.				
65	3. Interprofessional Collaboration<br>Description: Collaborating across professions in spiritual care				
66	4. Documentation of care<br>Description: Documenting Islamic spiritual care according to nursing standards.				
67	Competency - Attitude<br>Description: Students are able to demonstrate an empathetic and spiritually supportive presence.				
68	1. Presence<br>Description: Accompanying the patient completely, physically and spiritually				
69	2. Cultural sensitivity<br>Description: Aware of the diversity of patients' spiritual-religious beliefs				

NO	Element and sub elements model	1-3	4-9	7-9	comments
70	3. Islamic Ethics<br>Description: Students demonstrate morals and ethical values in practice.				
71	4. Trust<br>Description: Students are able to foster a sense of security and trust in patients.				

Second Round Delphi Questionnaire

Instructions for Completing the Delphi Questionnaire

Activity Objective

This questionnaire aims to establish consensus on the elements of the Islamic Spiritual Care learning model for undergraduate nursing students, based on the perspectives of experts, nursing practitioners, and professional students through two Delphi rounds.

Instructions

Participants are requested to evaluate each sub-element presented in this questionnaire according to its level of relevance and importance.

Please use a 9-point Likert scale (1–9) with the following criteria:

- **Scores 1–3**: Irrelevant / Not important
- **Scores 4–6**: Less relevant / Uncertain
- **Scores 7–9**: Highly relevant / Very important

Participants are encouraged to provide their ratings objectively, based on their knowledge, expertise, and professional experience.

Notes

- There are no right or wrong answers.
- Your responses are highly valuable in achieving group consensus.
- All responses will be kept confidential and used solely for research purposes

sub-elements	1-3	4-9	7-9	comments
course placement, and format				
learning style				
